# Collargolum in Cerebrospinal Meningitis

**Published:** 1905-08

**Authors:** 


### ABSTRACTS.

### Collargolum in Cerebrospinal Meningitis.

Gustav Bjorkman, M.D., professor of physiology, Milwaukee Medical College, in "An Index of Diseases," says: In my own experience I have met with great success in meningitis from the combined treatment with silver inunctions (Credé) and antiseptic packs on the skull. In some instances the suppuration took an outlet through the nose and ears; all cases that recovered regained their normal health in a remarkably short time. The method is to rub in from 1 to 3 grams (15 to 45 grains) of unguentum Credé twice a day at first and, when improvement follows, once a day. It is rubbed either on the trunk or inside the thigh for forty-five minutes. At the same time the hair of the scalp is closely clipped and moist borated gauze, drenched in a 1½ per cent. solution of formaldehyde, acetanilid and 1-10 per cent. of kresamine continually kept over the whole head, from the root of the nose backward down to the neck and on the sides, leaving the ears free. This pack is removed as soon as it becomes dry and a new one applied. Over the whole is placed a cap of oilcloth. There is no objection to the simultaneous use of an ice-cap on top of the oil-cloth or an ice-coil on the spine. It should be tried without hesitation, as it is entirely harmless, although in the beginning it causes inconvenience. By this combined treatment I have seen many cases in every type of meningitis recover,—cases which, I am sure, would otherwise have succumbed.—*Merck's Archives,* January, 1905.

